# To the editor regarding article “Coadministration of DPP-4 inhibitor and insulin therapy does not further reduce the risk of cardiovascular events compared with DPP-4 inhibitor therapy in diabetic foot patients: a nationwide population-based study”

**DOI:** 10.1186/s13098-018-0394-6

**Published:** 2018-12-17

**Authors:** Florian J. Mayer, Gerfried Pesau, Gerit H. Schernthaner

**Affiliations:** 1 Department of Laboratory Medicine, Medical University of Vienna, Vienna, Austria; 2 Division of Angiology, Department of Internal Medicine II, Medical University of Vienna, Vienna, Austria

With great interest we read the article by Lin et al. [1] about the effect of insulin and DPP4i therapy on the development of major adverse cardiovascular events in patients with diabetic foot syndrome. In this retrospective study clinical data of 19,791 patients with confirmed diabetic foot syndrome were obtained from the Taiwan National Health Insurance program. The patient population was stratified in three groups: those receiving insulin therapy, those receiving DPP4i inhibitors and those who received the combined therapy with insulin and DPP4i inhibitors. These groups were then investigated for the occurrence of a major adverse cardiovascular event between 2007 and 2014. In cox regression analysis (adjusted for age, sex, hypertension, hyperlipidemia, nephropathy, retinopathy, peripheral neuropathy, antithrombotic therapy and the index date) the group which was treated only with DPP4i inhibitors had a significantly better cardiovascular outcome than those who received the combined therapy (HR 0.55). No significant difference was found between the combined group and the insulin group for MACE. Based on these findings the authors conclude: “there was no additional benefit in reducing the risk of cardiovascular events by adding insulin to DPP4i-based therapy for the patients with diabetic foot”.

Insulin therapy is a cornerstone therapy for many patients with type 2 diabetes [2, 3]. Initial treatment of type 2 diabetes should ideally start with the modification of life-style factors and, if insufficient, should be followed by the initiation of oral anti-diabetic drugs. In the case of persistent hyperglycemia on (additional) oral anti-diabetic agents as well as in patients with initially very high plasma glucose levels, insulin remains the only option left for these patients. These are typically patients with advanced type 2 diabetes with increased insulin resistance and impaired peripheral insulin sensitivity, respectively, which has been repeatedly associated with adverse cardiovascular outcome and a high mortality rate [4, 5]. It is therefore unsurprising that the cardiovascular outcome of patients with insulin therapy is worse than of those who solely take anti-diabetic drugs. Attributing the increased risk of these patients entirely to the intake of DPP4i (instead of insulin) without at least adjusting for fasting plasma glucose and hbA1c in the multivariable regression analysis, is not convincing. On the contrary, the different outcomes of the groups investigated, are likely not caused by the different therapeutics, but rather by the degree of insulin resistance and impaired peripheral insulin sensitivity. Finally, the simple retrospective stratification of the patient population, without careful consideration of insulin dosing and glycemic control, does not provide enough evidence to allow any recommendation for the treatment of patients with diabetic foot syndrome. On a side note, the groups in figure 4 appear to be interchanged.
